# A three wave longitudinal study of school innovation climate and entrepreneurship teachers’ acceptance to technology: Moderating role of knowledge sharing and knowledge hiding

**DOI:** 10.3389/fpsyg.2022.1028219

**Published:** 2022-10-10

**Authors:** Rudsada Kaewsaeng-on, Suhaib Khaled AL-Takhayneh, Farooq Ahmed Jam, Bang-Lee Chang, Mahir Pradana, Saqib Mahmood

**Affiliations:** ^1^Faculty of Humanities and Social Sciences, Prince of Songkla University, Hatyai, Thailand; ^2^Councelling Psychology, Department of Guidance and Special Education, Faculty of Educational Sciences, Mutah University, Karak, Jordan; ^3^Global Illuminators, Kuala Lumpur, Malaysia; ^4^Department of Architecture and Urban Design, College of Environmental Design, Chinese Culture University, Taipei, Taiwan; ^5^Faculty of Communication and Business, Telkom University, Bandung, Indonesia; ^6^Faculty of Management Studies, International Islamic University, Islamabad, Pakistan

**Keywords:** school innovation climate, knowledge hiding, knowledge sharing, educational technology, teachers acceptance to technology

## Abstract

Entrepreneurship and business school teachers must extensively use technological and innovative tools to increase the efficacy of their instructional methods. This research aimed to investigate the teachers’ acceptance of technology in the school innovation climate, to enhance the use and effectiveness of educational technology in Thai entrepreneurship and business schools. Furthermore, the conditional influence of knowledge hiding and sharing on the link between school innovation climate and educational technology has been investigated and reported. Using a longitudinal study design data were gathered from the 204 entrepreneurship teachers of six different universities in Pattani, Bangkok, and Songkla Provinces, Thailand. Based on SamrtPLS 3.3.3 analysis, results revealed that the “school innovation climate” positively impacts educational technology use. Additionally, knowledge hiding and sharing moderated the relationship between “school innovation climate” and acceptance of educational technology (actual use of educational technology, perceived usefulness, and perceived ease of educational technology). Current research attempted to bridge the gap between knowledge management and innovation theories application in entrepreneurship education. The study brings key policy implications for school leaders and practitioners and suggests several directions for future research.

## Introduction

The modern educational evolution period has resulted in a transformation in teacher training and student educational development due to the integration of information technology into the educational system, especially in entrepreneurship education (Ismail et al., 2022). The advancement of technology has ushered in a new era in which digital teaching techniques have supplanted traditional ways of instruction (Buabeng-Andoh, 2019). The usage of digital gadgets is no longer a strange concept, and educators have even adopted it as a habit to execute instruction more effectively. Teachers may also vary their teaching approaches by using digital resources given by the state authority and information accessed from the internet. The advancement of information technology does not end there since technology has made the educational system more accessible and ubiquitous. Educational technology is increasingly popular and receiving an overwhelmingly positive reaction from educators of all ages and socioeconomic backgrounds (Ahmad-Ur-Rehman et al., 2010; AL Khuja and Mohamed, 2016; Leem and Sung, 2019).

Students’ acquisition of technological knowledge is mostly the responsibility of instructors, who must be able to not only utilize information technology to execute the learning process but also to build a digital learning environment for their students to flourish in (Ovcharuk et al., 2022). Teachers may encourage an innovative environment by providing technology settings that are conducive to it and that reward those who do so (Mumford, 2000; Ismail et al., 2022).

To be effective in twenty-first-century education, recommendations to build new ideas and techniques that instructors may adopt and implement should constantly be made (Agyei and Voogt, 2014). Novel viewpoints, techniques, course design, and educational technology help instructors improve their teaching performance (Prasojo et al., 2019) and enhance the actual use of technology in the school environment. Teachers’ use of technology makes it easier for students to access learning materials, and schools that have a sufficient number of IT tools may be able to increase students’ enthusiasm for learning (Chou et al., 2019). Furthermore, instructors’ engagement and role in creative teaching might encourage their students to get their own life experience and knowledge through the actual use of technology (Habibi et al., 2020). These techniques used by teachers encourage an innovative climate and are referred to as “innovation climates” or “climates for innovation” (Chen and Hou, 2016). The current study contends that a “school innovation climate” is necessary to promote educational technology usage in educational institutions.

Throughout the 1990s, there was a substantially increased awareness of knowledge management in various organizational contexts. Business, computer science, economics, and sociology are just a few of the domains in which knowledge sharing and knowledge transfer have been extensively investigated. Even though many people have talked about knowledge sharing in organizations (Srivastava et al., 2006; Tohidinia and Mosakhani, 2010; Tangaraja and Rasdi, 2013; Wu et al., 2022), few studies have looked into how employees hide their knowledge (Peng, 2013; Černe et al., 2014; Ahmad and Akbar, 2021; Chatterjee et al., 2021; Mahmoud et al., 2021; Waheed et al., 2021). Although more emphasis is being paid to the “knowledge sharing” behaviors of workers in various working contexts, limited information is available about academics’ “knowledge hiding” activities in the academic environment. As Mohayidin et al. (2007) stated, when it comes to achieving the country’s goal of establishing a knowledge-based society in Thailand, universities are focused on producing competent graduates with intellectual and problem-solving abilities and social knowledge. The primary purpose of establishing academic institutions is to share knowledge (Kidwell et al., 2000). Considering the importance of knowledge hiding and knowledge sharing in school innovation climate, this study inducted knowledge hiding and knowledge sharing as a moderator on the influence of school innovation climate on educational technology. The studies focusing on teachers acceptance to technology in entrepreneurship and business schools are scarce in literature. Specially, the researches on innovation climate and linking it to knowledge hiding and sharing behaviors as well as its role in determining the technology acceptance by teachers is a unique area of study. Current research attempts to bridge this research gap by providing fresh empirical evidence from Thai business and entrepreneurship schools.

Newman et al. (2020) devised a direction for the prospective investigation that identifies opportunities for researchers to go theoretically and empirically to enhance literature on innovation climate in different settings. A recent study on entrepreneurship intention among ASEAN nations also included a Thai sample, along with other ASEAN nations, based on the theory of planned behavior (Virasa et al., 2022). ASEAN nations’ dynamics and growing economic conditions have created ample opportunities for emerging entrepreneurs. Based on ASEAN economic community vision 2025, all nations, including Thailand, are paying extra attention to mushrooming entrepreneurship education in the country (Itakura et al., 2017). A report on entrepreneurship activities in Thailand and Indonesia by GEM has reported that total early-stage entrepreneurial activity among the adult population was 14.1 to19.7% (Bosma and Kelley, 2019; Virasa et al., 2022). This shows the enormous potential of entrepreneurship in the Thai setting. Thus making this study more relevant and advancing the literature in contextual terms. Another recent study in the Thai context attempted to develop a Thailand entrepreneurial spirit index and recommended further investigating the parameters that may sketch out the innovation climates of Thai entrepreneurial education and knowledge-related behaviors (Tripopsakul et al., 2022). Thus responding to such a call for an investigation, current research further extended the literature by studying the currently proposed framework in the Thai educational context. This study extends the literature on the innovation climate in the universities of Pattani, Bangkok, and Songkla provinces of Thailand. It is thus relevant to investigate the impact of school innovation climate, knowledge hiding, and knowledge sharing on educational technology, as shown by the present research. The present research, which is grounded on the “technology acceptance model (TAM), by Davis (1989)” provides theoretical grounds for phenomenon under investigation in this research. It provided some linkages how knowledge sharing and knowledge hiding behaviors may play role in technology acceptance among Thai business school teachers (Davis and Venkatesh, 1996). The application of TAM in school innovation climate of Thai business schools in itself is a theoretical advance pitched by current research. Thus, current study seeks to provide answers to the following crucial research questions:

- Does school’s innovative climate favorably influence the educational technology acceptance among business and entrepreneurship school teachers?
- Does knowledge hide and sharing moderate the relationship between the school’s innovation climate and educational technology?

## Literature review

### Teachers’ preferences and technology acceptance model

Teachers’ opinions regarding emerging technologies certainly affect their judgments concerning whether or not to employ new technology in the classroom. This study is mainly grounded in the TAM (Davis, 1989). According to this theory, one’s actual usage of a technological system is explicitly or implicitly impacted by the perceived usefulness of the technology, and the perceived ease of the technology in connection to external factors, attitudes, and behavioral intentions impact the actual usage of a technological system. Based on this theory, teachers’ acceptance of technology in the “school innovation climate” impact the “actual use of educational technology,” “perceived usefulness of educational technology,” and “perceived ease of educational technology.” Further, this study considers knowledge hiding and knowledge sharing as the external factors of behavioral intentions, which influence the school innovation climate to increase or decrease the education technology usage in Thai entrepreneurship and business schools.

### School innovation climate and educational technologies

Innovative teaching behavior is defined as the deliberate actions of instructors, particularly pre-service teachers, who deliberately attempt to incorporate educational technology within their instructional plans. The attempts include innovation, which impacts students’ willingness to invent new things. Teachers’ acceptance of technology in the school innovation climate covers acts that stimulate conceptions and everyday behavior *via* technology (Hornstra et al., 2015; Sofwan et al., 2021). Teachers’ technology acceptance, on either side, has been described as technology adoption in a specific sense (Teo, 2014; Chou et al., 2019), which includes teachers’ hold of positive inclinations toward technology, willingness to use technology, awareness of the usefulness of technology, and control over the technology used in classroom instruction. The actual use of technology is influenced by teachers’ readiness to use technology in the teaching and learning process in a school innovation climate and will bring the perceived usefulness and ease of educational technology (Ovcharuk et al., 2022). It has also been shown that instructors with a positive attitude toward new teaching approaches, such as technology-infused education, are more likely to gain knowledge or learn new things (Nikolopoulou and Gialamas, 2016).

In the last few years, scholars have started to look into how innovative climates affect people’s behaviors (Mazhar et al., 2012; Waheed et al., 2012; Bamberger, 2018; Kurniawan and Managi, 2018; Machrus and Desmita, 2019) and enhance the use of technology. An innovation climate is characterized as “shared perceptions at the team or organizational level regarding the extent to which team or organizational processes encourage and enable innovation” (Begley et al., 2006). A recent study investigated the benefits of innovation and referred that innovation brings ease to integrating educational technology in teaching activities and teachers’ consciousness about the advantages of technology for innovative teaching (Sofwan et al., 2021). When the performance expectancy between innovation and behavior improves, adopting teaching practices more compatible with innovation behavior becomes more viable (Abdullah and Ward, 2016; Waheed et al., 2017; Abbas et al., 2021; Subasinghe, 2021). Some previous studies also found the positive impact of “school innovation climate” on motivation, work attitude and commitment, and technology (Jaiswal and Dhar, 2015; Lee and Idris, 2017). The present research extended the literature by investigating the positive influence of “school innovation climate” on educational technologies.

Kang et al. (2016) suggested that an innovation climate encourages exploring new dimensions. Based on the TAM model, this study considers the “school innovation climate” to influence the “actual use of educational technology, perceived usefulness of educational technology, and perceived ease of educational technology.” Based on theoretical support from the TAM model and literary evidence from past research, it is hypothesized that;

H1: *There is a positive relationship between the school innovation climate and the educational technologies*, i.e., *(a) actual use of educational technology*, *(b) perceived usefulness of educational technology*, *and (c) perceived ease of educational technology.*

### Role of knowledge hiding as a moderator

There could be various aspects concerning knowledge hiding in educational institutions. From the standpoint of information concealing, hidden, or restricted knowledge is most often sought by those seeking it (Serenko and Bontis, 2016). Furthermore, according to the Conservation of Resource (COR) theory (Hobfoll, 1989), staff members of enterprises have a strong desire to protect and conserve their resources (knowledge). Their conduct becomes more challenging when they believe their hoarded resources are at risk of being taken away. They act like knowledge hiders at that period. Assuming that an organization has a good atmosphere for knowledge sharing and that the knowledge being hidden is not difficult, the motivation of those who hide knowledge might influence the organization’s inclination to conceal knowledge. This is the consequence desired by the knowledge hiders, which drives them to keep the resource hidden (knowledge).

As a result, when knowledge hiders are incentivized to conceal information, the knowledge-sharing culture of the business is negatively impacted. The same pattern holds for individuals who seek information to improve their job performance: if they are strongly driven to learn, they will be able to learn more and faster. A recent study on knowledge hiding recommended investigating it as a moderator between innovation antecedents and outcomes, especially among knowledge workers (Fauzi, 2022). Another recent research in the Chinese context was conducted on knowledge hiding and innovative behaviors and reported that knowledge hiding has the potential to play a moderating role in the innovation environment to hinder the success of any innovative activity (Chen et al., 2022). A recent study conducted in the Iranian context on knowledge hiding and IT-enabled work organizations also pointed toward a research gap and controversy about entrepreneurship education innovation climate and technology adoption in the context of knowledge hiders (Khan et al., 2016; Almulla, 2018; Andole et al., 2020; Al Muhaissen and Alobidyeen, 2022; Labafi et al., 2022). They pointed toward a huge research gap and suggested investigating the role of knowledge hiding in decreasing intentions for IT-enabled entrepreneurial activities. Thus current research attempted to bridge this gap by proposing the moderating role of knowledge hiding between school innovation climate and the use of educational technologies. The behaviors such as knowledge hiding has been considered negatively associated with technology acceptance (Davis and Venkatesh, 1996). Thus, presence of such vital constructs in real business school phenomenon has rarely been investigated in past research to focus on teachers’ acceptance to educational technology (Labafi et al., 2022). Thus current research attempted to bridge this gap by incorporating knowledge hiding as moderator between school climate and acceptance to technology.

Considering the “TAM,” external factors impact the actual use of technology. These external factors are the attitudinal and behavioral intentions of individuals. In this study, “knowledge hiding” is an external factor of behavioral intention to measure the influence of “school innovation climate” on educational technology. Further, individuals make decisions regarding the use of technology based on their behavioral intentions. As a result, this study hypothesizes:

H2: *Knowledge hiding moderate association of school innovation climate with educational technologies*, i.e., *(a) actual use of educational technology*, *(b) perceived usefulness of educational technology*, *and (c) perceived ease of educational technology.*

### Role of knowledge sharing as a moderator

The studies have concentrated on the significance of “knowledge sharing” in organizations (Dezi et al., 2021). While the effect of knowledge sharing is scarcely investigated in educational institutions, on the other hand, information sharing has been associated with increased consumer benefits and a shorter generation cycle (Ma et al., 2008), and enhancements that are possible (Papa et al., 2018; Santoro et al., 2020). Knowledge sharing may be an important part of the planning process for information management approaches (Abu-Saqer and Abu-Naser, 2019) and has been investigated at both the individual and organizational levels (Abu-Saqer and Abu-Naser, 2019; Dezi et al., 2021). When seen at the individual level, knowledge sharing is defined as the amount employees share the information they gain with their coworkers and other members of the business (Teh and Yong, 2011). These include publicly available knowledge that may be gathered and maintained in official documents and proprietary information that is hard to apply (Masih et al., 2021, 2022). It is impossible for employees to respond to highly important organizational concerns if they do not exchange knowledge with their coworkers (Ma et al., 2008) and, as a result, have little useful knowledge about themselves (Curado et al., 2017). Teachers’ acceptance of technology in the “school innovation climate” directly influence the educational technology in educational institutions (Laužikas and Miliūtė, 2020; Laužikas et al., 2021). Along with (Shipton et al., 2006), a “school innovation climate” empowers educational technology, and knowledge sharing plays a role in enhancing the impact of the “school innovation climate” on educational technology.

Thus current study aims to bridge the existing research gap in business and entrepreneurship literature by suggesting the interactive effects of knowledge sharing and school innovation climate on acceptance of educational technologies among teachers in business schools. Similar knowledge oriented behaviors such as knowledge sharing has been considered positively related with technology acceptance by teachers (Davis and Venkatesh, 1996; Curado et al., 2017). Hence, emergence of such important constructs in business and entrepreneurship school setting has scarcely been studied in past research related to teachers’ acceptance to educational technology (Santoro et al., 2020). Thus current research highlighted to bridge this research gap by proposing knowledge sharing as moderator between school climate and acceptance to technology.

Based on the “TAM,” external factors, attitudes, and behavioral intentions impact the actual usage of a technological system. This study incorporates “knowledge sharing” as an external factor of behavioral intention to measure the fostered influence of “school innovation climate” on educational technology. Further behavioral intentions influence the decision of individuals to utilize technology. Therefore, this study hypothesizes:

H3: *Knowledge sharing moderate association of school innovation climate with educational technologies*, i.e., *(a) actual use of educational technology*, *(b) perceived usefulness*, *and (c) perceived ease of educational technology. In the case of higher levels of knowledge sharing*, *the influence of school innovation climate and technology use will be enhanced.*

### Theoretical framework of the study

**Figure 1:**
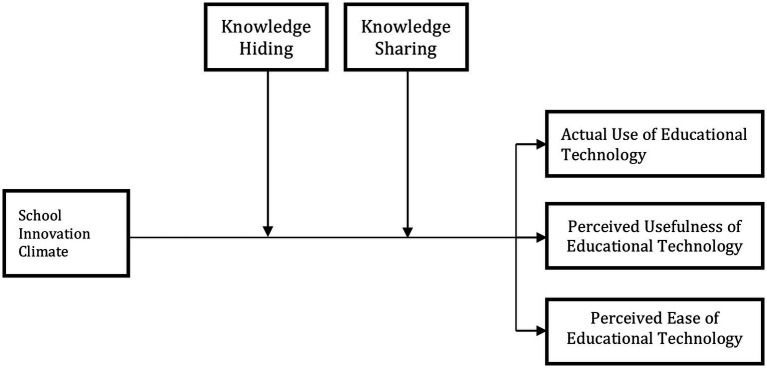
Theoretical framework of the study.

Using a survey of the previous studies and the TAM, the researchers established a conceptual framework for this research, as shown in Figure 1.

## Research methodology

The studies focusing on Thai business and entrepreneurship school teachers are scarce in the literature and this sample population has been rarely investigated in past literature related to business education (Liu, 2022). Thus considering on the population of this research adds value to contextual advance made by this research as previous studies on school innovation climate recommended to consider Thai school teachers’ attitudes and acceptability of technology (Kanawapee et al., 2022). Similarly, in business education context Thai society is collectivist society with high power distance (Hofsted, 1980). So attempting to investigate their knowledge hiding and knowledge sharing behaviors is an incremental to the body of knowledge.

The participants in this research are instructors from six public and private universities in Pattani, Bangkok, and Songkla provinces of Thailand. The data were collected using a “convenience sampling technique” using a “time-lagged approach with a three-wave survey.” The participants responded to the predictor variable (School Innovation Climate) at the first point of time (T1), two moderating variables (Knowledge Hiding and Knowledge Sharing) at the second point of time (T2), and three outcome variables (Actual Use of Educational Technology, Perceived Usefulness of Educational Technology, and Perceived Ease of Educational Technology) at the third point of time (T3). All three points of time (T1, T2, and T3) were set with a gap of 2 weeks. However, the convenience sampling method has been widely criticized in past literature and is controversial (Etikan et al., 2016)., several other studies also supported using this sampling technique where time-lagged and difficult data collection processes are involved (Jager et al., 2017; Kempen and Tobias-Mamina, 2022). Convenience sampling was the right approach for this investigation since it is a type of sampling where the first available observable is used for the investigation, and no requirement for more data streams to be gathered.

Additionally, it facilitates data collection more timely and cost-effectively, and inclusion is accessible to individuals. Dalle et al. (2021) articulated a series of steps that authors took after consent from their universities’ coordinators. After that, the management of universities in Bangkok, Pattani, and Songkla Provinces of Thailand was approached to seek formal permission for this research. One of the researcher’s universities’ ethics committees also obtained ethical approval. In-depth explanations of the research’s purpose were provided to relevant management of business and entrepreneurship schools. Upon gaining formal authorization and contact details, entrepreneurship instructors were contacted and inquired whether they would be willing to participate in a voluntary survey.

Teachers who consented to complete the survey were handed over the questionnaire to complete. At the first point of time (T1), 330 forms were handed over to rate the predictor variable, and the respondents returned 293. At the second point of time (T2), after 2 weeks, 293 forms were handed out again to the same participants for the rating of two moderating variables, and the respondents returned 242 at this time. At the third point of time (T3), which came after 2 weeks after the completion of second round, 242 forms were distributed again to the same participants to rate the three outcome variables, and the respondents returned 213. Researchers finalized the 204 questionnaire sets for the analysis, and 09 were excluded due to partially filled or unengaged responses. The difference between the number of participants from the start phase to the end constitutes the final response rate of 61.82%. The participants were given unique ID codes to recognize the questionnaires that would be compiled after the end of the final phase. The surveys were created in English since English is widely spoken and understood at higher educational institutions in Thailand.

### Measures of the study

To evaluate the association between “school innovation climate” and educational technology. The independent variable “school innovation climate” was assessed using a four-item scale developed by Fraser and Rentoul (1982). Respondents were invited to rate the score on a “5-point Likert scale ranging from 1 = strongly disagree to 5 = strongly agree.” Furthermore, the “Perceived Usefulness of Educational Technology” was assessed using a four-item scale developed by (Davis, 1989). To assess the “Perceived Ease of Educational Technology,” a four-item scale adapted from Alharbi and Drew (2014) was employed. In “Actual Use of Educational Technology,” a three-item scale (for each) was adapted from Mathieson et al. (2001) and Moon and Kim (2001) at the same time and used in conjunction with each other. The responses were measured at “a 5-point Likert-type scale (1 = strongly disagree, 5 = strongly agree).” The moderator variable “knowledge hiding” was assessed using eight items on “a five-point Likert scale ranging from ‘not at all to a great extent,” which was derived from Connelly et al. (2012). Another moderator variable, knowledge sharing, was measured using the six-item scale developed by Lu et al. (2006), on “a 5-point Likert-type scale (1 = strongly disagree, 5 = strongly agree).”

## Data analysis and results

### Demographic characteristics of the respondents

Table 1 describes the details of the demographic of participants who voluntarily participated in this research. The analysis of one way ANNOVA was performed to check the influence of demographic variables on study outcomes. It was revealed that only qualification and teaching experience has a significant influence on outcomes. So these two demographic variables were controlled during further analysis.

**Table 1:** Respondents’ demographic characteristics.

Variables			Teachers (%)
Gender	Female		47.30
	Male		52.70
Age	18–25 years		13.20
	26–30 years		27.40
	31–35 years		39.10
	36 and above		20.30
Qualification/Degree level	Undergraduate level		–
	MBA/MS/Graduate level		33.2
	Ph.D./Post-graduate		55.5
	Post doc		11.3
Teaching experience	1–5 years		9.20
	6–10 years		36.50
	11–15 years		31.40
	16 years and above		22.90

This study used the Software SmartPLS 3.3.3 for preliminary evaluation and analysis of constructs’ reliability and validity. The results demonstrated that the “school innovation climate” positively impacted the use of educational technologies in educational institutions in Thailand.

### Measurement model assessment

**Figure 2:**
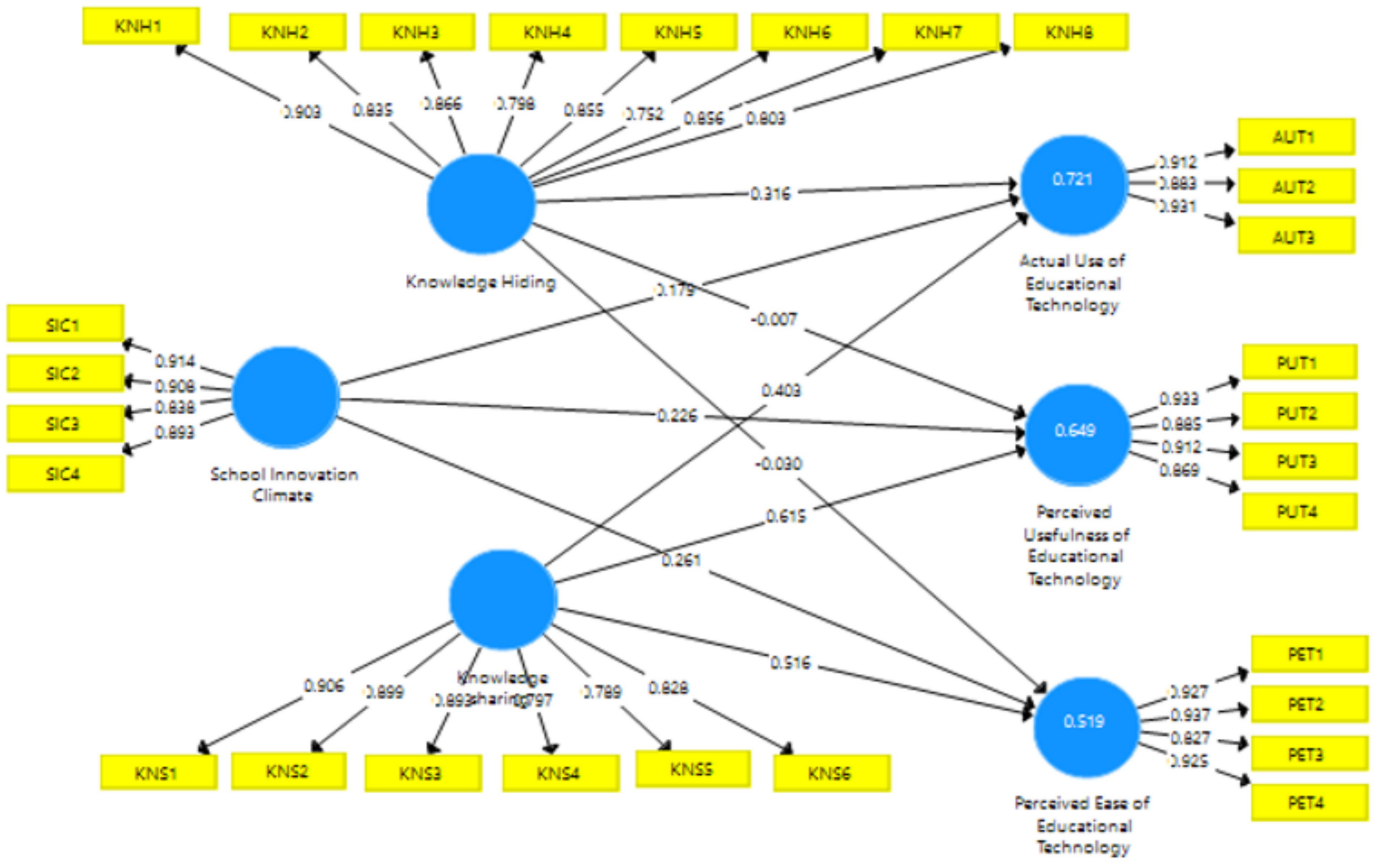
Measurement model.

The validation test is performed to assess a measuring scale’s consistency. As explained in Figure 2 above to assess the validity of the data, “Confirmatory Factor Analysis (CFA)” was performed, which is meant to confirm the most dominant factors in a group of variables by examining the relationships between them (factor loading). “When a standardized factor loading (SFL) of more than 0.70 is found in an indicator, it is considered to have strong validity” (Hair et al., 2019). These results are reported in Table 2 by the findings of outer loading, which has reached the threshold point.

**Table 2:** Outer loadings.

	AUT	KNH	KMS	PET	PUT	*SIC*
AUT1	0.912					
AUT2	0.883					
AUT3	0.931					
KNH1		0.903				
KNH2		0.835				
KNH3		0.866				
KNH4		0.798				
KNH5		0.855				
KNH6		0.752				
KNH7		0.856				
KNH8		0.803				
KNS1			0.906			
KNS2			0.899			
KNS3			0.893			
KNS4			0.797			
KNS5			0.789			
KNS6			0.828			
PET1				0.927		
PET2				0.937		
PET3				0.827		
PET4				0.925		
PUT1					0.933	
PUT2					0.885	
PUT3					0.912	
PUT4					0.869	
SIC1						0.914
SIC2						0.908
SIC3						0.838
SIC4						0.893

This study tested the “validity and reliability of the constructs” by using “convergent validity, which includes “Cronbach’s Alpha (CA), rho_A, Composite Reliability (CR), and Average Variance Extracted (AVE)” (Henseler et al., 2015). “Cronbach’s alpha and rho_A” are recommended to be more than 0.7. The “Composite Reliability (CR)” of a variable is determined by a group of indicators that indicates whether or not the variable has strong “Composite Reliability (CR),” defined as higher than 0.7. According to the proposed method, the determined value of “Average Variance Extracted (AVE)” should be higher than 0.50. Table 3 depicts that all the figures meet the threshold point; as a result, “convergent validity” has been established (Hair et al., 2017a; Hair et al., 2019).

**Table 3:** Construct reliability and validity.

	Cronbach’s alpha	rho_A	CR	AVE
Actual use of educational technology	0.894	0.894	0.934	0.826
Knowledge hiding	0.938	0.943	0.948	0.697
Knowledge sharing	0.925	0.931	0.941	0.728
Perceived ease of educational technology	0.928	0.960	0.948	0.820
Perceived usefulness of educational technology	0.922	0.927	0.945	0.810
School innovation climate	0.911	0.924	0.938	0.790

CR, composite reliability; AVE, average variance extracted.

SEM includes the term “discriminant validity” to verify that a measurement of a construct is both experimentally exclusive and capable of explaining observed events that other measurements in the framework appear unable to explain (Hair et al., 2010). Subsequently, “discriminant validity” requires that “a test does not correlate too highly with measures from which it is supposed to differ” (Campbell, 1959). Campbell (1981) approach was used to determine the questionnaire’s discriminant validity. According to this criteria, “the square root of the AVE greater than the sum of all correlations within the same row and column of the specified construct,” as seen in Table 4 below.

**Table 4:** Fornell and Larcker.

	AUT	KNH	KNS	PET	PUT	*SIC*
Actual use of educational technology	0.909					
Knowledge hiding	0.800	0.859				
Knowledge sharing	0.818	0.841	0.853			
Perceived ease of educational technology	0.797	0.615	0.706	0.905		
Perceived usefulness of educational technology	0.773	0.692	0.796	0.712	0.900	
School innovation climate	0.767	0.806	0.825	0.663	0.728	0.889

“The Fornell and Larcker (1981) criteria,” which are the most frequently used “discriminant validity criterion,” are ineffective in particular situations (Henseler et al., 2009; Rönkkö and Evermann, 2013), denoting that the quite commonly used “discriminant validity yardstick” may have a shortcoming (Rönkkö and Evermann, 2013). Henseler et al. (2015) have developed a novel strategy for determining “discriminant validity” that they feel is superior to the current approaches. “The Heterotrait-Monotrait Correlations Ratio (HTMT)” is a novel method for determining “discriminant validity.” To ensure that all research constructs are unique, the HTMT ratio was set below 0.90. Table 5 shows that all results are below the HTMT criterion of 0.85.

**Table 5:** Heterotrait-Monotrait ratio.

	AUT	KNH	KNS	PET	PUT	*SIC*
Actual use of educational technology						
Knowledge hiding	0.763					
Knowledge sharing	0.793	0.799				
Perceived ease of educational technology	0.746	0.629	0.720			
Perceived usefulness of educational technology	0.749	0.736	0.758	0.741		
School innovation climate	0.737	0.762	0.790	0.682	0.787	

Before evaluating the proposed structural model, it is recommended that the multi-collinearity test be applied to the constructs under consideration. In the presence of collinearity, it is hard to determine the influence of a single variable on the outcome. This research aimed to investigate the usage of variance inflation factors, often known as VIFs, in the examination of multi-collinearity. The evaluation of VIF is based on using two thresholds, “VIF <3 and < 5.” The criterion of 3 is more cautious, but the threshold of 5 is common and acceptable since there is no multi-collinearity issue across the constructs (Hair et al., 2019). The results of VIF for current study data are presented in Table 6.

**Table 6:** Variance inflation factor (inner VIF).

	AUT	KNH	KNS	PET	PUT	*SIC*
Actual use of educational technology						
Knowledge hiding	3.947			3.947	3.947	
Knowledge sharing	4.340			4.340	4.340	
Perceived ease of educational technology						
Perceived usefulness of educational technology						

The term “goodness of fit (GoF)” has been coined to describe how well a model fits the data in a PLS-SEM setting. On the other hand, the “goodness of fit” measurement should not be used as a goodness of fit metric in any study since it cannot reliably distinguish valid from invalid models because its utility is limited to certain model settings. To produce approximation fit indices such as “SRMR and NFI,” the results of a PLS-SEM model estimate are taken into consideration, as are the values of these parameters that satisfy a certain threshold “(for example, SRMR 0.08 and NFI > 0.90).” The goodness of fit of this model has been shown in line with Table 7, 8 below.

**Table 7:** Goodness of fit.

	Saturated model	Estimated model
SRMR	0.086	0.092
d_ULS	3.226	3.690
d_G	7.804	7.976
Chi-Square	1326.368	1346.076
NFI	0.922	0.914

**Table 8:** Goodness of fit.

	*R* ^2^	*R*^2^ adjusted
Actual use of educational technology	0.421	0.405
Perceived ease of educational technology	0.519	0.492
Perceived usefulness of educational technology	0.649	0.629

While doing data analysis, the phrase “coefficient of determination” is a sophisticated notion based on statistical modeling. “coefficient of determination” is a statistical term that illustrates how two variables linked together might influence one another’s variance. This score varies from 0.0 to 1.0, with 1.0 indicating a perfect fit and, as a result, a highly trustworthy model for future forecasts, and 0.0 shows that the model does not adequately describe the data at all (i.e., the model fails to describe the data adequately). According to Hair et al. (2019), “the coefficient of determination was symbolized as R2 which is the reflection of the variable quality included in the model, and criterion determined R2 value ≥0.670 as substantial, 0.330 as moderate, and 0.190 as weak.” The present study presents 42.1, 64.9, and 51.9% variance in Actual Use of Educational Technology, Perceived Usefulness of Educational Technology, and Perceived Ease of Educational Technology, which depicts moderate.

### Structural model assessment

#### Path coefficients

**Figure 3:**
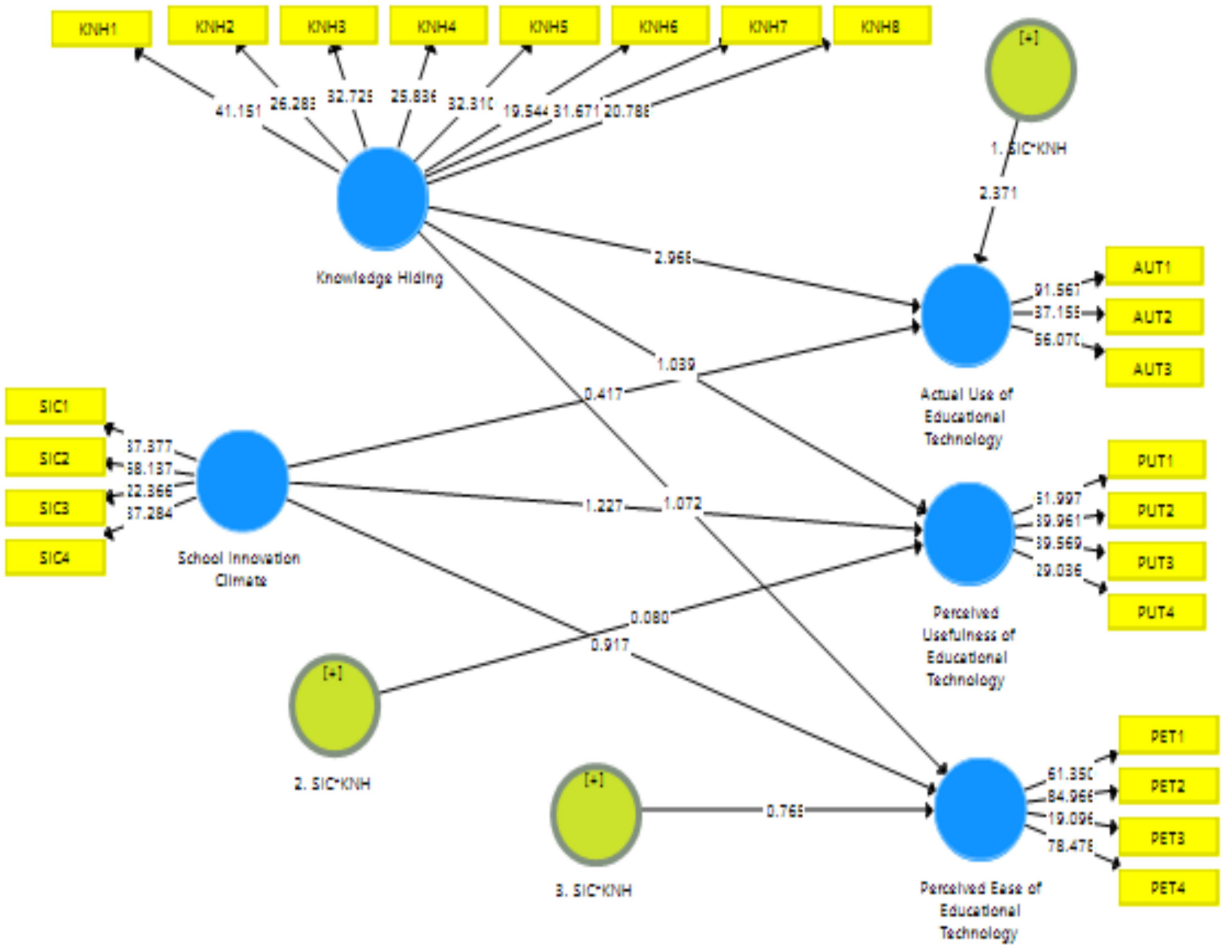
Structural model 2.

As presented in Figure 3, the path coefficients are clearly evident in structural model 1 and structural model 2. To investigate the causal linkages between the elements impacting educational technology, the researchers evaluated the structural model used in this research. Many factors to measure statistical significance may be used to assess if the data support the hypotheses, including path coefficients (β), t-values, and *p*-values (Hair et al., 2017a). To create the statistics that would be used to determine statistical significance, a bootstrapping procedure with resampling of 5,000 was used in the SmartPLS 3.3.3 program (Hair et al., 2017b).

#### Hypothesis testing (direct effect)

To evaluate H1a, b, and c, we first examined the direct influence of independent variables on dependent variables. The findings of the direct connection between variables are shown in the following table. The present research established a statistically significant positive association between the “school innovation climate” and “actual use of educational technology (Coefficient = 0.600, *p* = <0.05), perceived usefulness of educational technology (Coefficient = 0.485, *p* = <0.05), and perceived ease of educational technology (Coefficient = 0.481, *p* = <0.05).” Additionally, Table 9 presents the findings of the direct relationship hypotheses H1a, b, and c, indicating that all hypotheses were accepted.

**Table 9:** Direct relationships.

Hypothesis		Original sample	Sample mean	*t*-statistics	*P*-values	Supported
H_1a_	*SIC* - > AUT	0.600	0.566	2.650	0.008	Yes
H_1b_	*SIC* - > PUT	0.485	0.464	3.079	0.002	Yes
H_1c_	*SIC* - > PET	0.481	0.461	2.815	0.005	Yes

SIC, school innovation climate; AUT, actual use of educational technology; PUT, perceived usefulness of educational technology; PET, perceived ease of educational technology.

#### Hypothesis testing (moderation)

**Figure 4:**
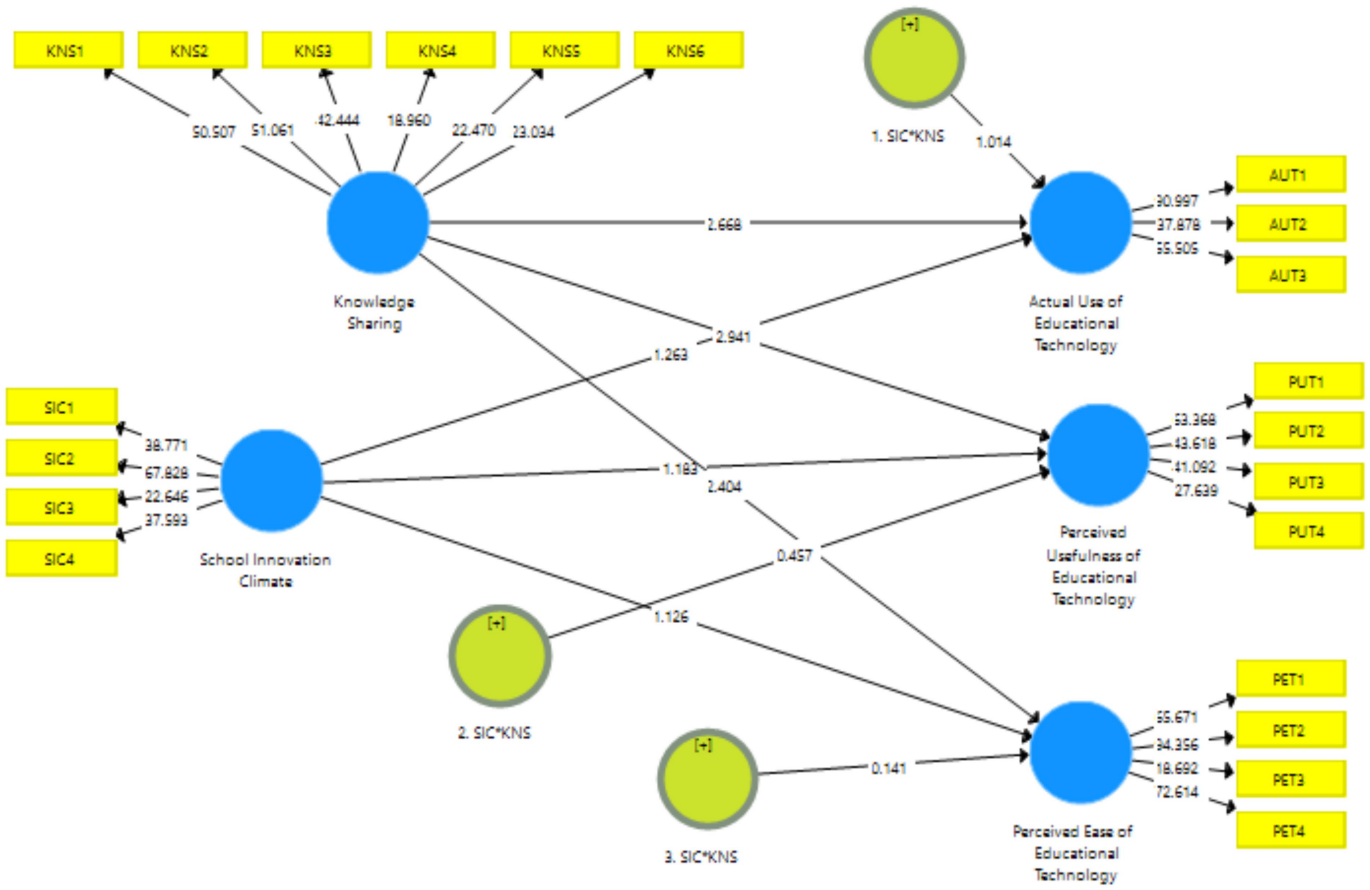
Structural model 1.

Using the SmartPLS 3.3.3 program, the authors tested the moderation between independent and dependent variables, as shown in Figures 3, 4. Results in Table 10 illustrates that knowledge hiding moderates the relationship between the “school innovation climate” and “actual use of educational technology (Coefficient = 0.506, *p* < 0.05), perceived usefulness of educational technology (Coefficient = 0.442, *p* < 0.05), perceived ease of educational technology (Coefficient = 0.454, *p* < 0.05).” Results in Table 11 illustrates that knowledge sharing moderates the relationship between the “school innovation climate” and “actual use of educational technology (Coefficient = 0.493, *p* = <0.05), perceived usefulness of educational technology (Coefficient = 0.462, p = <0.05), perceived ease of educational technology (Coefficient = 0.451, *p* = <0.05).” Moreover, the results of the first moderation hypotheses H2a, b, and c are presented in Table 10, reflecting that all hypotheses were accepted. The results of the second moderation hypotheses, H3a, b, and c, are presented in Table 11, depicting that all hypotheses are accepted.

**Table 10:** Moderation analysis 1.

Hypothesis		Original sample (O)	Sample mean (M)	*t*-statistics (|O/STDEV|)	*P*-values	Supported
H2a	SIC*KNH - > AUT	0.506	0.481	3.319	*0.001*	Yes
H2b	SIC*KNH - > PUT	0.442	0.416	4.447	*0.000*	Yes
H2c	SIC*KNH - > PET	0.454	0.451	3.559	*0.000*	Yes

SIC, school innovation climate; AUT, actual use of educational technology; PUT, perceived usefulness of educational technology; PET, perceived ease of educational technology; KNH, knowledge hiding.

**Table 11:** Moderation analysis 2.

Hypothesis		Original sample (O)	Sample mean (M)	*t*-statistics (|O/STDEV|)	*P*-values	Supported
H3_a_	SIC*KNS - > AUT	0.493	0.469	2.817	0.000	Yes
H3_b_	SIC*KNS - > PUT	0.462	0.436	3.743	0.000	Yes
H3c	SIC*KNS - > PET	0.451	0.413	3.168	0.000	Yes

SIC, school innovation climate; AUT, actual use of educational technology; PUT, perceived usefulness of educational technology; PET, perceived ease of educational technology; KNS, knowledge sharing.

## Discussion

### Findings

The research discovered that teachers’ acceptance of technology in school innovation climate encourages the implementation educational technology in entrepreneurship and schools, a major finding from study results. These circumstances provide evidence that instructors are well prepared to face the challenges of today’s educational environment by incorporating technology features into their classrooms. Instructors formerly employed technology like computers or LCD projectors to instruct students in labs or advanced rooms outfitted with information and communication technology facilities. However, the school innovation climate has provided an entirely different approach, in which educational technology may take place anywhere, independent of the exact location. These results demonstrate that teachers are prepared to support educational technology acceptance in schools.

The first hypothesis of this research proposed a positive association between school innovation climate and teachers’ acceptance to educational technology. The present research established a statistically significant positive association between the “school innovation climate” and “actual use of educational technology (Coefficient = 0.600, *p* = <0.05), perceived usefulness of educational technology (Coefficient = 0.485, *p* = <0.05), perceived ease of educational technology (Coefficient = 0.481, *p* = <0.05).”

These findings are in line with Perienen (2020), which supports the acceptance of technology by consumers. According to the findings, instructors stress in this section of the survey that teachers who utilize educational technology may create a more meaningful learning experience for their pupils. These findings are also supported by the findings of Waruwu et al. (2020), which demonstrate that a creative atmosphere favors behavior and outcomes.

The study’s findings reveal that, in addition to the direct relationships between school innovation climate and educational technologies, knowledge hiding and knowledge sharing have a contingent influence on the link between “school innovation climate” and educational technologies. According to current research results, knowledge hiding reduces the influence of the “school innovation climate” on educational technologies. Results illustrates that knowledge hiding moderates the relationship between the “school innovation climate” and “actual use of educational technology (Coefficient = 0.506, *p* < 0.05), perceived usefulness of educational technology (Coefficient = 0.442, *p* < 0.05), perceived ease of educational technology (Coefficient = 0.454, *p* < 0.05).” These findings are consistent with those of Chatterjee et al. (2021), where knowledge hiding is proved to be a constraint for performance. Results also revealed that knowledge sharing moderates the relationship between the “school innovation climate” and “actual use of educational technology (Coefficient = 0.493, *p* = <0.05), perceived usefulness of educational technology (Coefficient = 0.462, *p* = <0.05), perceived ease of educational technology (Coefficient = 0.451, *p* = <0.05).” Hence, the results of this study show that knowledge sharing helps the school innovation climate to enhance the use of educational technology, consistent with the results of the previous studies by Alkhazali et al. (2021).

### Theoretical implications

There are several academic implications of the current study, which is based on the TAM, that are worthy of discussion, and there are various reasons why the current study is incremental to the literature. The teachers’ acceptance of technology in the “school innovation climate” in predicting and enhancing educational technology among universities in Pattani, Songkla, and Bangkok provinces of Thailand, has been investigated for the first time in this study. As per the author, none of the previous studies has investigated these constructs in a given context. In addition, this study makes a major contribution by identifying the contingent role of knowledge hiding and sharing in the relationship between school innovation atmosphere and educational technologies. Specifically, this study contributes significantly to current knowledge by illustrating that knowledge sharing improves the “school innovation climate,” which helps to increase “Actual Use of Educational Technology, Perceived Usefulness of Educational Technology, and Perceived Ease of Educational Technology.” Thus, current research advanced by integrating the TAM model with knowledge management theories and providing a new avenue for future theoretical integration between knowledge management and technology acceptance theories. The current study is important to incorporate three different domains of research together in the single comprehensive framework. It paved a way for future studies of TAM with school innovation climate and education governance literature. Similarly, TAM with knowledge hiding and knowledge sharing is another area of future exploration for scholars. Thus current research opened several avenues for future theoretical explorations in the fields of strategy, organizational behavior and technology acceptance domains and inclusiveness of these domains with business and entrepreneurship literature. This study is among the earliest to test the moderating role of knowledge sharing and knowledge hiding to provide evidence from a unique Thai cultural context.

### Practical implications

Academicians and professionals in higher education institutions and members of the organizational community will benefit significantly from the findings of this study. With an emphasis on the positive influence of a “school innovation climate” on educational technologies should develop criteria for selecting instructors who accept technology in a school innovation climate. The instructor is critical in successfully incorporating new technologies into educational environments (Teo, 2011; Leem and Sung, 2019). Teachers’ attitudes about new technology have an impact on how new technology is used in the classroom for educational purposes (Carver, 2016; Leem and Sung, 2019). Consequently, instructors’ perceptions about technology are important determinants in teaching and learning environments. STEM learning is highly regarded at the moment, and many colleges and universities focus on incorporating new technology in their programs (Solanki and Xu, 2018; Dalle Grave, 2020). Apart from these critical areas, higher education institutions may pursue specialized development programs in relevant fields such as educational technology.

The current research brings several policy insights for Thai business and entrepreneurship schools for motivating teachers for accepting technology. There is a strong need for training interventions to improve knowledge sharing and knowledge hiding among business schools’ teachers in Thailand. Additionally, several other factors such as impact of their technology attitude on their teaching outcomes may also be considered. Specially, if these factors are studies on Thai actual entrepreneur sample would bring more useful insights for practitioners and entrepreneurs.

Moreover, educational institutions must consider the surrounding atmosphere to encourage knowledge sharing and discourage knowledge hiding. Yet the outcomes of this analysis demonstrated that knowledge sharing, along with a “school innovation climate,” might increase the use of educational technology. Finally, the education policy-making bodies may encourage the establishment of educational technologies and knowledge sharing across educational institutions using training and other motivational interventions.

### Limitations and future research directions

Throughout the current study several flaws should be considered in future studies. First and foremost, the participants in the current study were solely university lecturers from business and entrepreneurship schools in the Thai provinces of Pattani, Songkla, and Bangkok. No additional subjects and faculties were included in the list to increase generalizability among Thai higher education institutions. Other Thai areas, on the other hand, may be investigated in future studies for a larger and more inclusive sample. Accordingly, a comparative study might be conducted to establish the effect of “school innovation climate” on educational technologies from different provinces in Thailand, with the findings being compared for better generalizability.

In contrast, the influence of “school innovation climate” on a range of educational technology is likely to be investigated at the secondary and middle school levels in future studies. As a final point, the current study used a time-lagged methodological approach, in which data was collected from participants at three distinct periods separated by 2 weeks and then pooled. In the future, ongoing longitudinal studies may be conducted to eliminate the typical technique bias and boost the universal applicability of the results. Comparative studies in a regional context, like among ASEAN countries, European countries, and Asian as well as Gulf nations, may bring several key insights for policy development in this area.

## Conclusion

The current research has focused on its objectives to explore the theoretical linkages between school innovation climate and technology acceptance by Thai business and entrepreneurship school teachers. Additionally, the interactive effects of knowledge hiding, knowledge sharing with school innovation climate were proposed and empirically tested for its impact of teachers’ acceptance to technology. The rigorous longitudinal methodology was followed to collect data and for determining better causal relationships among the study constructs proposed in theoretical framework. The data was further analyzed by PLS (SEM) and bootstrapping method was also applied to test generalizability of findings on larger data sets. The results revealed that school innovation climate has positive association with all three factors of technology acceptance. Additionally, the moderating role of knowledge hiding and knowledge sharing was also confirmed by study results. In case of higher levels of knowledge sharing behaviors the acceptance to technology was more as compared to normal associations. Similarly, higher levels of knowledge hiding provided psychological hurdle in technology acceptance by business and entrepreneurship teachers in Thailand. The study provided several practical and theoretical implications for practitioners, school managers, entrepreneurs and business professionals as well as economic policy makers.

## Data availability statement

The raw data supporting the conclusions of this article will be made available by the authors, without undue reservation.

## Author contributions

RK-o helped in data collection and data analysis. SA-T helped in idea development, theory building, and literature review. FJ helped in data interpretation and methodology design and execution. B-LC helped in writing results section. MP helped in writing discussion and implications. SM helped in literature review, introduction, and proof editing of complete article. All authors contributed to the article and approved the submitted version.

## Conflict of interest

The authors declare that the research was conducted in the absence of any commercial or financial relationships that could be construed as a potential conflict of interest.

## Publisher’s note

All claims expressed in this article are solely those of the authors and do not necessarily represent those of their affiliated organizations, or those of the publisher, the editors and the reviewers. Any product that may be evaluated in this article, or claim that may be made by its manufacturer, is not guaranteed or endorsed by the publisher.
